# The lncRNA H19/miR-541-3p/Wnt/β-catenin axis plays a vital role in melatonin-mediated osteogenic differentiation of bone marrow mesenchymal stem cells

**DOI:** 10.18632/aging.203267

**Published:** 2021-07-26

**Authors:** Hui Han, Tingyu Tian, Guoqian Huang, Dalu Li, Shimao Yang

**Affiliations:** 1 Department of Center of Pediatric Dentistry, Jinan Stomatology Hospital, Jinan 250001, Shandong Province, China; 2 Department of Oral and Maxillofacial Surgery, Jinan Stomatology Hospital, Jinan 250001, Shandong Province, China

**Keywords:** H19, osteogenic differentiation, adipogenic differentiation, melatonin, miR-541-3p

## Abstract

Implant dentures become the first choice for denture restoration in patients with tooth loss. However, oral implants often fail in osteoporosis (OP) patients. Melatonin (MT) induces osteogenic differentiation of bone mesenchymal stem cells (BMSCs), suggesting its therapeutic potential in OP treatment. Long non-coding RNA H19 induces osteogenic differentiation of BMSCs, while its regulatory mechanism in MT-involved osteogenic and adipogenic differentiation of BMSCs remains elusive. Ovariectomized (OVX) rat was used to construct an OP model, and bone quality was assessed. Meanwhile, the expression of H19, miR-541-3p, MT and adiponectin (APN) was examined by quantitative reverse transcription-PCR (qRT-PCR) or ELISA. The adipogenic and osteogenic differentiation of BMSCs were determined by oil red O staining and alizarin red S staining, respectively. The targeting relationships between H19, miR-541-3p and APN mRNA were predicted by bioinformatics and confirmed by RNA immunoprecipitation and dual-luciferase reporter assay. The results showed that MT, H19 and APN were down-regulated, while miR-541-3p was up-regulated in the OVX rat model. At the cellular level, MT reduced adipogenic differentiation, heightened osteogenic differentiation of BMSCs, and activated Wnt/β-catenin pathway, which were reversed by the MT2 selective inhibitor 4-P-PDOT. Overexpressing H19 facilitated the osteogenic differentiation and inhibited the adipogenic differentiation of BMSCs mediated by MT, while H19 knockdown or overexpressing miR-541-3p had the opposite effect. Moreover, H19 functioned as a competitive endogenous RNA and sponged miR-541-3p, and miR-541-3p targeted APN. Overall, MT modulates the osteogenic and adipogenic differentiation of BMSCs by mediating H19/miR-541-3p/APN axis, providing a new reference for the targeted therapy of OP.

## INTRODUCTION

Implant dentures have gradually become the first choice for denture restoration for patients with tooth loss due to their advantages such as no damage to natural teeth, small size, comfort, stability, and good chewing function. They are known as the third set of teeth of humans. However, in patients with osteoporosis (OP), oral implants often fail due to the lack of initial stability and the inability to form good osteosynthesis [1, 2]. The risk of OP in women is usually higher than that in men, and ovarian aging and estrogen deficiency are the main causes of postmenopausal OP (PMOP) [3]. Osteogenic differentiation plays a critical role in maintaining the skeletal microenvironment balance, and mesenchymal stem cells (MSCs) can differentiate into multiple cell types, including osteoblasts, chondrocytes, and lipoblasts. Therefore, enhancing osteogenic differentiation of bone mesenchymal stem cells (BMSCs) is essential for improving OP treatment [4, 5].

Melatonin (MT), a methoxyindole, is previously found mainly synthesized and secreted by the pineal gland at night under normal light and dark conditions [6]. Recently, melatonin is confirmed to be synthesized in the mitochondria, suggesting that every cell can synthesize melatonin, including bone marrow mesenchymal stem cells or osteoblasts. [7]. In addition to circadian rhythm, MT also has effects of antioxidants [8], anti-aging [9], neurodegenerative disease resistance [10], and immune regulation [11], etc. Besides, MT has significant effects on apoptosis, angiogenesis, tumor suppression and anti-proliferation of various tumor cells [12]. Notably, MT is implicated in the homeostasis of bone metabolism, and MT reduction is a key factor in bone loss and OP [13–15]. Moreover, MT enhances osteogenic differentiation of MSCs by regulating the Wnt/β-catenin, AMPK/β-catenin, and other signaling pathways [16, 17]. The MT injection into rats induces the expression of osteogenesis-related genes in BMSCs, promotes osteoblast differentiation, and elevates the bone mineral density (BMD), bone volume fraction (BV/TV), and trabecular number (Tb.N) in the OP model [18]. It is suggested that MT can be used to treat OP. However, the downstream signaling molecular mechanism of MT's involvement in modulating osteogenic and adipogenic differentiation of BMSCs remains to be further explored.

Long non-coding RNAs (lncRNAs) are greater than 200 nt in transcript lengths and do not have protein-coding functions [19]. LncRNAs are confirmed to mediate osteogenic differentiation of BMSCs by emerging studies. For example, lncRNA MEG3 abates the osteogenic differentiation of BMSCs in PMOP by regulating miR-133A-3p [20]. LncRNA NEAT1 promotes osteogenic differentiation of human BMSCs (hBMSCs) by modulating miR-29b-3p/BMP1 [21]. LncRNA H19 is a member of the lncRNA family, and studies have shown that up-regulated H19 facilitates osteogenic differentiation of BMSCs by facilitating stromal cell-derived factor 1 (SDF-1) through miR-149 [22]. Besides, H19 strengthens osteogenic differentiation of BMSCs by regulating the miR-140-5p/SATB2 axis [23]. Thus, H19 is a key gene in bone diseases, while whether it plays a role in MT-mediated differentiation of BMSCs remains unclear.

MicroRNAs (miRNAs), as a kind of single stranded noncoding RNA with a length of about 22 nt, are one of the important regulatory targets of lncRNAs [24]. miR-541-3p, as one member of multiple miRNAs, has been found to inhibit bone metastasis of prostate cancer [25]. And it represses osteogenic differentiation and is expected to become a potential target for regulating bone formation [26]. Adiponectin (APN) is an adipocyte-specific factor initially reported in 1995, which plays an important role in obesity, diabetes, inflammation, atherosclerosis and cardiovascular diseases [27]. The latest studies have shown that APN stimulates bone formation and promotes osteogenic differentiation of BMSCs through Wnt/β-catenin [28]. However, the miR-541-3p-APN axis in MT-mediated differentiation of BMSCs needs further investigation.

Here, we discovered that H19 was down-regulated in the OP rat model. *In vitro*, the H19 level in MT-treated BMSCs was significantly increased, and overexpressing H19 enhanced the promotion of MT-mediated osteogenic differentiation and the inhibition of adipogenic differentiation. Besides, bioinformatics analysis showed that H19 could competitively inhibit miR-541-3p, which targeted adiponectin (APN). Moreover, overexpression of H19 reduced the miR-541-3p level and elevated the APN expression. Therefore, we speculated that MT modulated BMSC differentiation by regulating the H19-miR-541-3p-APN axis, thus playing a key pharmacological role in OP.

## RESULTS

### H19 and APN were down-regulated, while miR-541-3p was up-regulated in OP rats

**Figure 1:**
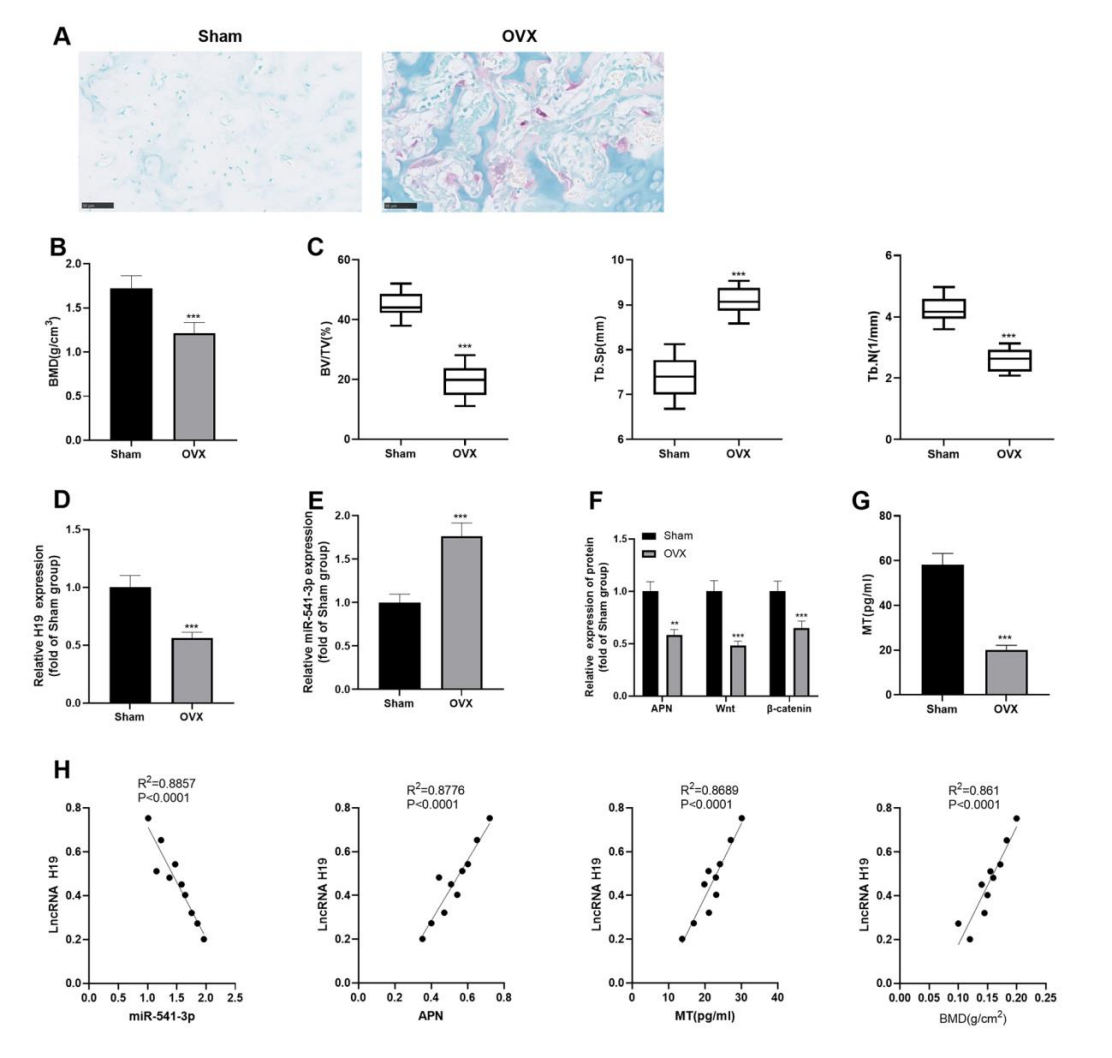
**H19 and APN were down-regulated, while miR-541-3p was up-regulated in OP rats.** (**A**) TRAP staining was performed to label osteoclasts. Scale: 50 μm. (**B**, **C**) Expression of BMD, BV/TV, Tb. SP and Tb.N in mandibular tissues of the OP rat model. (**D**, **E**) The levels of H19 and miR-541-3p in mandibular tissues of the OP rat model were examined by qRT-PCR. (**F**) WB was implemented to verify the protein levels of APN and Wnt/β-catenin in mandibular tissues of the OP rat model. (**G**) MT content in the caudal vein of the OP rat model was detected by ELISA. *** *P* < 0.001 (vs. the Sham group). (**H**) Person linear regression analysis was used to determine the correlation between H19 and miR-541-3p, APN, MT and BMD. *R*^2^ = 0.8857, *P* < 0.0001; *R*^2^ = 0.8776, *P* < 0.0001; *R*^2^ = 0.8689, *P* < 0.0001; *R*^2^ = 0.8610, *P* < 0.0001. Data were presented as mean ±SEM (n =10) and analyzed using one-way analysis of variance.

SD rats were purchased and the OP rat model was established by ovarian extraction. First, we labeled osteoclasts by using the TRAP staining to verify the modeling. The results indicated that there was a significant increase in osteoclast markers in the OVX group (vs. the Sham group) (Figure 1A). Then, we measured the BMD and Tb.Th of the rats' mandibles. As a result, the rats in the OVX group had significant bone loss, decreased BMD, BV/TV and Tb.N, and elevated Tb.Sp (Figure 1B, 1C). Mandibular bone tissues and caudal vein blood of the rats were collected. qRT-PCR, Western blotting, and ELISA were conducted to detect the profiles of H19, miR-541-3p, and APN in bone tissues and the MT expression in the caudal vein. As shown in Figure 1D–1G, in the OVX group, H19 and APN were down-regulated, while miR-541-3p was up-regulated in the mandibular bone tissue, and the MT content in the blood decreased (vs. the Sham group). In addition, we analyzed the correlation of various molecules with Pearson and found that H19 was reversely related to miR-541-3p and positively correlated with MT, APN, and BMD in mandibular bone tissues (Figure 1H). The above results suggested that H19, miR-541-3p, MT and APN were involved in the progression of OP.

### MT inhibited the adipogenic differentiation and facilitated the osteogenic differentiation of BMSCs

**Figure 2:**
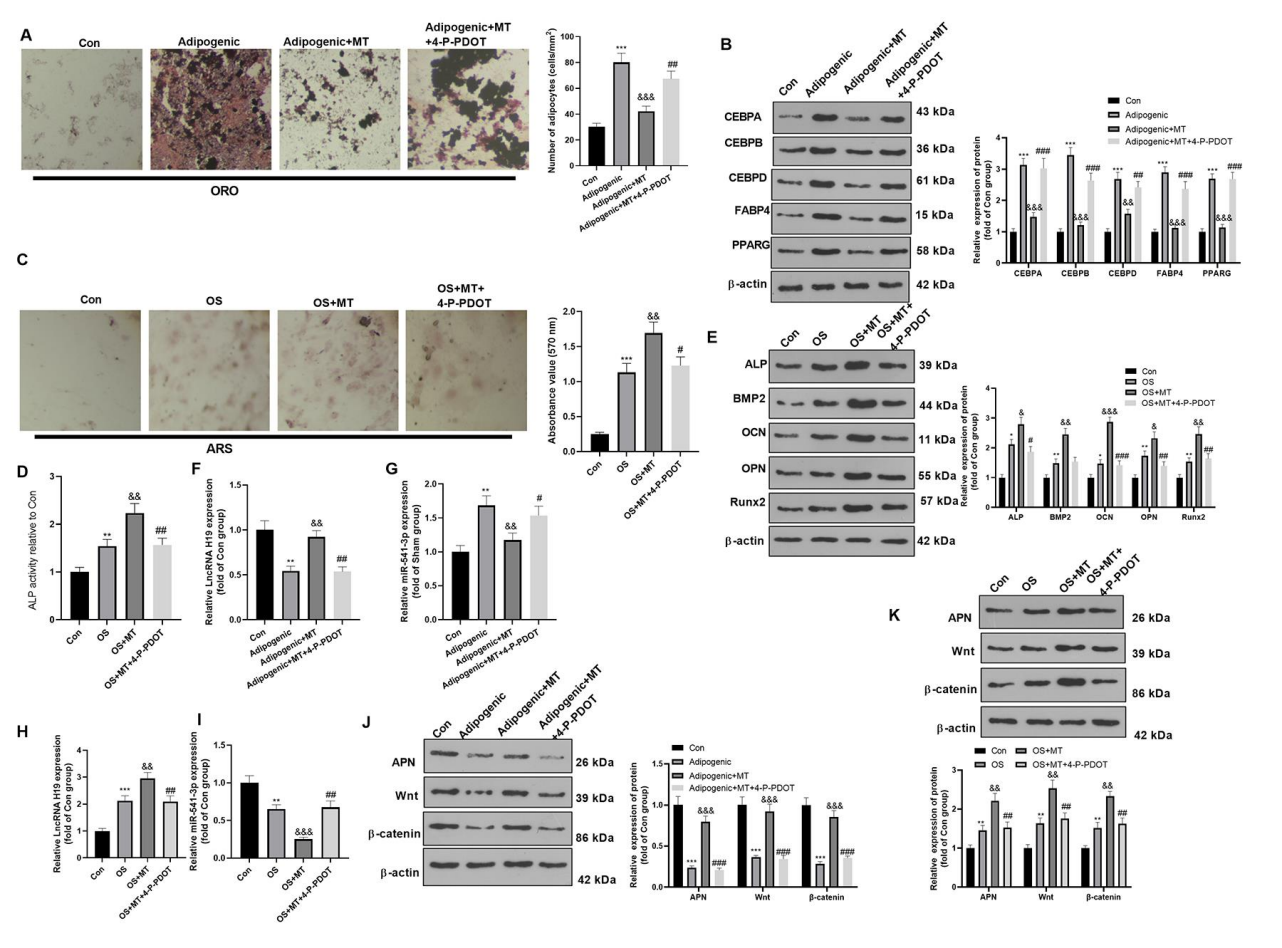
**MT inhibited the adipogenic differentiation and facilitated the osteogenic differentiation of BMSCs.** BMSCs were treated with 100 μM MT and/or MT2 selective inhibitor 4-P-PDOT (1 μg/ml). (**A**) BMSCs were cultured in adipogenic differentiation culture medium. The adipogenic differentiation of BMSCs was tested by ORO staining. Scale bar: 200 μm. (**B**) The expression of adipocyte-related proteins (including CEBPA, CEBPB, CEBPD, FABP4, and PPARG) after MT/4-P-PDOT treatment in BMSCs was analyzed by WB. (**C**) ARS activity test was conducted to evaluated the osteogenic differentiation of BMSCs. Scale: 200 μm. (**D**) The ALP activity was detected using ALP activity test kit. (**E**) The relative expression of osteogenic proteins (including ALP, BMP2, OCN, OPN and Runx2) was analyzed by WB. (**F**–**I**) The relative expression of H19 and miR-541-3p in BMSCs was analyzed by qRT-PCR. (**J**, **K**) WB was conducted to analyze the levels of APN and Wnt/β-catenin after MT treated BMSCs. *P<0.05, **P<0.01, ***P<0.001 (vs. Con group), &*P*>0.05, &&*P*<0.01, &&&*P*<0.001 (vs. OS group). #*P*>0.05, ##*P*<0.01, ### *P* < 0.001 (vs. Adipogenic+MT group). Data were presented as mean ±SEM (n =3) and analyzed using one-way analysis of variance.

We investigated the influence of MT on the osteogenic potential of BMSCs and its potential mechanism. First, the BMSCs were isolated, differentiated into osteogenic/adipogenic cells, and treated with MT (10 μmol/L) with or without MT2 inhibitor 4-P-PDOT (1 μg/ml). Then, we analyzed the impact of MT on the adipogenic differentiation of BMSCs. ORO staining confirmed that MT treatment inhibited the formation of lipid droplets in BMSCs after 16 days of adipogenic differentiation (compared with Adipogenic group, Figure 2A), while 4-P-PDOT treatment enhanced the formation of lipid droplets in BMSCs (compared with Adipogenic+MT group, Figure 2A). Additionally, Western blotting results confirmed that MT significantly reduced the profiles of adipogenesis-related genes in BMSCs, including CEBPA, CEBPB, CEBPD, FABP4, and PPARG (compared with Adipogenic group, Figure 2B). In comparison to the Adipogenic+MT group, the addition of 4-P-PDOT promoted those proteins (Figure 2B). Subsequently, we examined the influence of MT on osteoblastic differentiation of BMSCs. ARS staining and ALP activity assay showed that MT facilitated osteoblastic differentiation of BMSCs, resulting in a significant elevation in the mineralized matrix and ALP activity (Figure 2C, 2D). Besides, we conducted Western blotting to monitor the levels of ALP, BMP2, OCN, OPN and Runx2, which were typical markers of osteoblastic differentiation. As a result, the levels of these four markers were heightened after MT treatment (vs. the OS group) (Figure 2E). Interestingly, the BMSCs dealt with 4-P-PDOT had less osteoblastic differentiation, lower expression of ALP, BMP2, OCN, OPN and Runx2, and less ALP activity (compared with OS+MT group, Figure 2C–2E). Finally, the impact of MT on the expression of H19, miR-541-3p, APN and Wnt/β-catenin pathway was probed. The results illustrated that H19, APN and Wnt/β-catenin pathway were downregulated in BMSCs after Adipogenic differentiation, while miR-541-3p was promoted (compared with Con group, Figure 2F, 2G, 2J). However, H19, APN and Wnt/β-catenin pathway were downregulated in BMSCs after OS differentiation, while miR-541-3p was promoted (compared with Con group, Figure 2H, 2I, 2K). MT enhanced the H19 expression, decreased the miR-541-3p expression, and facilitated the profiles of APN and Wnt/β-catenin (compared with Adipogenic group or OS group, Figure 2F–2K). However, 4-P-PDOT treatment repressed H19, APN and Wnt/β-catenin pathway, and promoted miR-541-3p expression (Figure 2F–2K). Overall, these results manifested that MT enhanced osteogenic differentiation of BMSCs.

### Overexpressing H19 enhanced the osteogenic effect of MT on BMSCs

**Figure 3:**
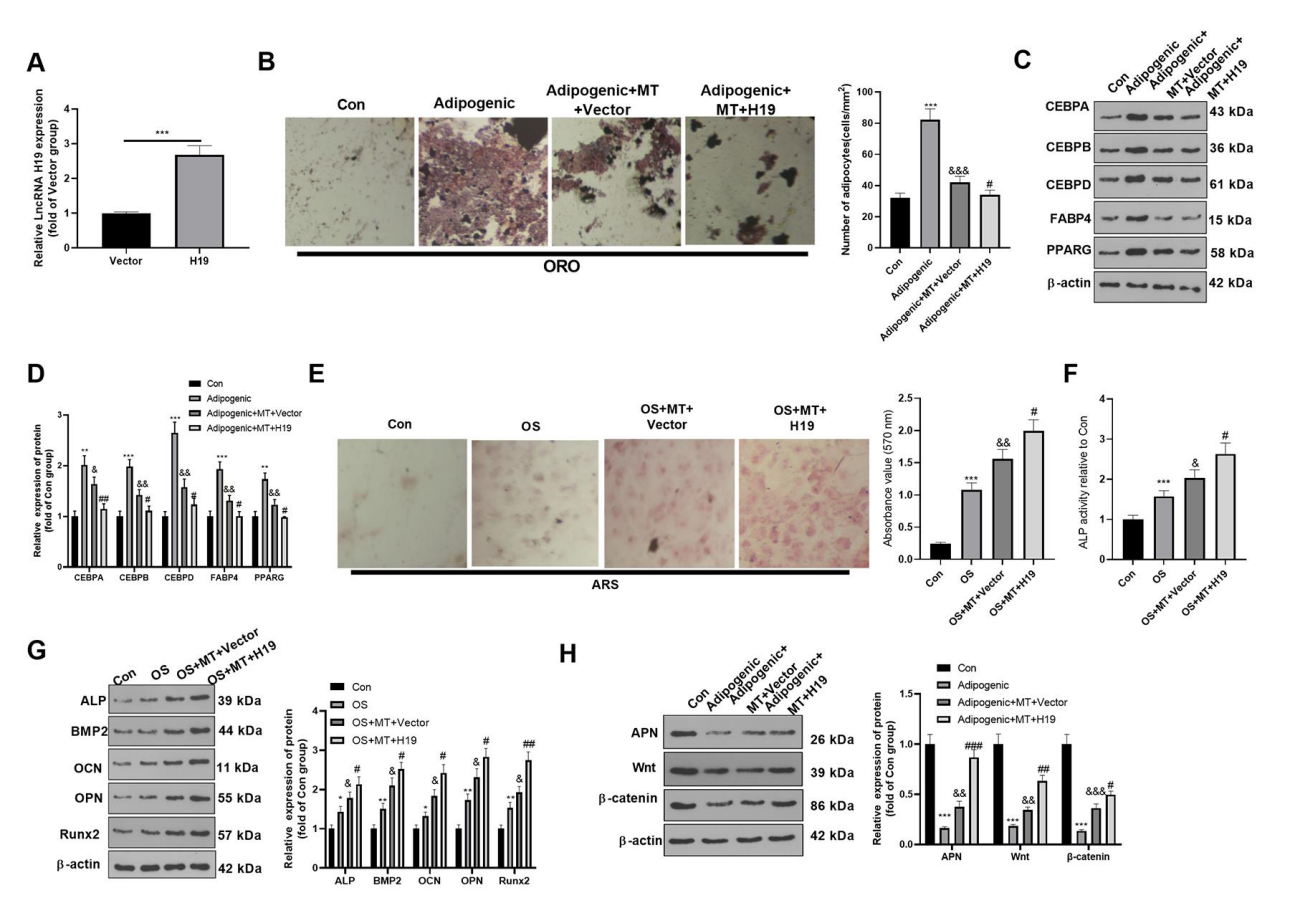
**Overexpressing H19 enhanced the osteogenic effect of MT on BMSCs.** BMSCs were transfected with H19 overexpression plasmids or vector, and then cultured in Adipogenic/OS differentiation culture medium. (**A**) Expression of H19 in BMSCs after transfection with H19 overexpression plasmids was detected by qRT-PCR. (**B**) ORO staining verified the role of H19 in adipogenic differentiation of BMSCs. Scale: 200 μm. (**C**, **D**) The expression of adipocyte-related proteins (including CEBPA, CEBPB, CEBPD, FABP4, and PPARG) in BMSCs was analyzed by WB. (**E**) ARS activity test was conducted to evaluated the osteogenic differentiation of BMSCs. Scale: 200 μm. (**F**) The ALP activity was detected using ALP activity test kit. (**G**) The relative expression of osteogenic proteins (including ALP, BMP2, OCN, OPN and Runx2) was analyzed by WB. (**H**) WB was utilized to analyze the protein levels of APN/Wnt/β-catenin in BMSCs cultured in adipogenic differentiation culture medium. *P<0.05, **P<0.01, ***P<0.001(vs. Con group), &*P*>0.05, &&*P*<0.01, &&&*P*<0.001 (vs. Adipogenic/OS group), #*P*<0.05, ##*P*<0.01, ###*P*<0.01 (vs. Adipogenic/OS+MT+Vector group). Data were presented as mean ±SEM (n=3) and analyzed using one-way analysis of variance.

It is known from the above studies that MT increases the H19 expression in BMSCs, but its role remains unclear. Therefore, we transfected the H19 overexpression plasmid and its negative vector in BMSCs to probe the role of overexpressing H19 on adipogenic/osteogenic differentiation of BMSCs. qRT-PCR demonstrated that H19 was overexpressed in BMSCs after the transfection of the H19 overexpression plasmids (Figure 3A). ORO staining showed that up-regulation of H19 inhibited lipid droplet formation in BMSCs (Figure 3B). Besides, H19 overexpression significantly reduced the expression of CEBPA, CEBPB, CEBPD, FABP4, and PPARG (Figure 3C, 3D). Moreover, ARS staining and ALP activity detection revealed that the up-regulation of H19 further increased the number of mineralized nodules and ALP activity (Figure 3E, 3F). Western blotting manifested that ALP, BMP2, OCN, OPN and Runx2 were up-regulated after H19 up-regulation compared with the OS+MT+Vector group (Figure 3G). Finally, we probed the influence of H19 on the expression of APN and Wnt/β-catenin pathway under Adipogenic differentiation, and it was found that up-regulating H19 facilitated the APN and Wnt/β-catenin expression (compared with Adipogenic +MT+Vector group, Figure 3H). Therefore, the above data confirmed that enhancing the H19 expression reduced the Adipogenic differentiation and promoted osteogenic differentiation of BMSCs.

### H19 knockdown mitigated the osteogenic differentiation of MT-treated BMSCs

**Figure 4:**
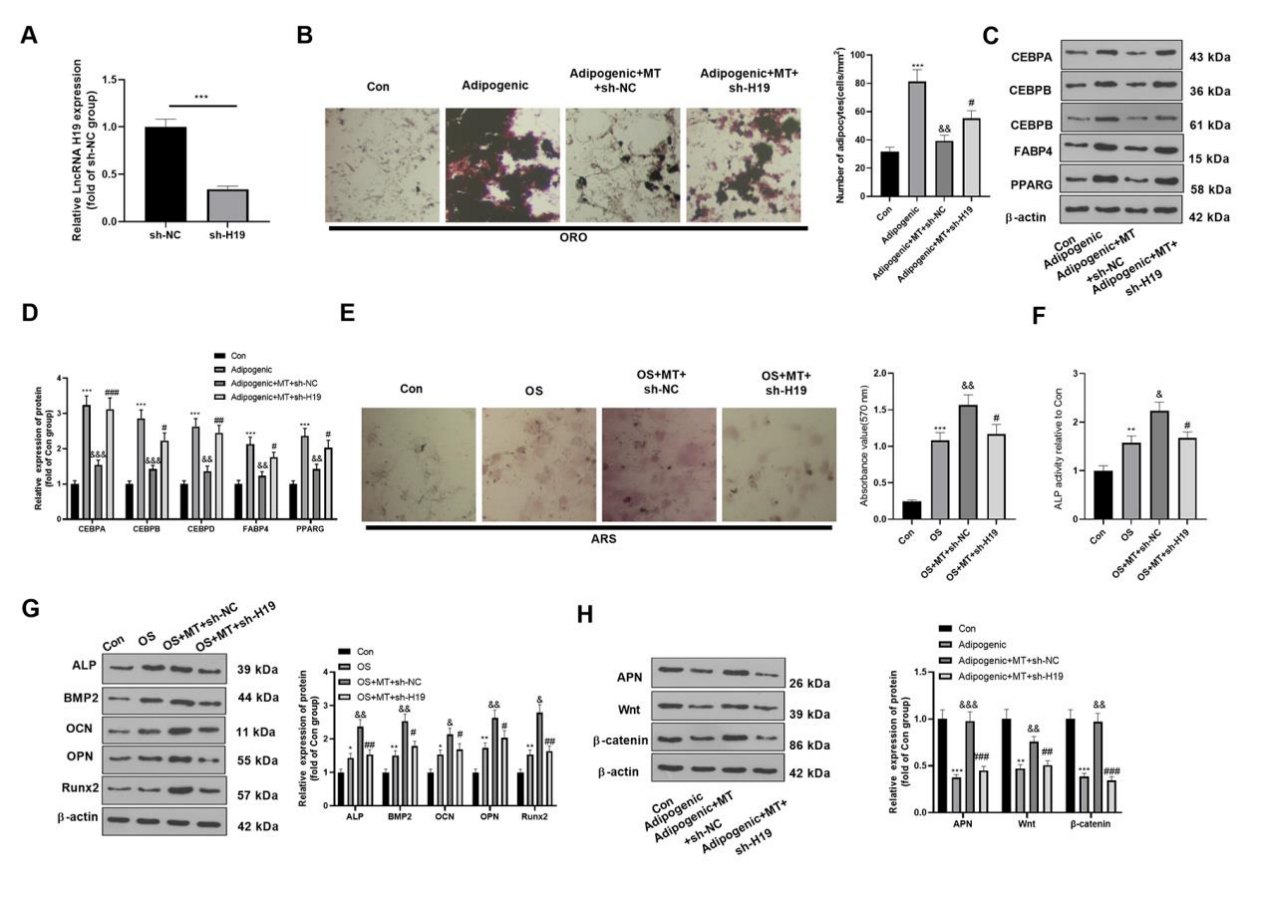
**Downregulating H19 repressed the osteogenic effect of MT on BMSCs.** BMSCs were transfected with sh-H19 or sh-NC, and then cultured in Adipogenic/OS differentiation culture medium. (**A**) Expression of H19 in BMSCs after transfection with H19 overexpression plasmids was detected by qRT-PCR. (**B**) ORO staining verified the role of H19 in adipogenic differentiation of BMSCs. Scale: 200 μm. (**C**, **D**) The expression of adipocyte-related proteins (including CEBPA, CEBPB, CEBPD, FABP4, and PPARG) in BMSCs was analyzed by WB. (**E**) ARS activity test was conducted to evaluated the osteogenic differentiation of BMSCs. Scale: 200 μm. (**F**) The ALP activity was detected using ALP activity test kit. (**G**) The relative expression of osteogenic proteins (including ALP, BMP2, OCN, OPN and Runx2) was analyzed by WB. (**H**) WB was utilized to analyze the protein levels of APN/Wnt/β-catenin in BMSCs cultured in adipogenic differentiation culture medium. *P<0.05, **P<0.01, ***P<0.001(vs.Con group), &*P*>0.05, &&*P*<0.01, &&&*P*<0.001 (vs. Adipogenic/OS group), #*P*<0.05, ##*P*<0.01, ###*P*<0.01 (vs. Adipogenic/OS+MT+sh-NC group). Data were presented as mean ±SEM (n=3) and analyzed using one-way analysis of variance.

To further confirm the role of H19 on MT-treated BMSCs, we transfected a H19-knockdown model on BMSCs using sh-H19. qRT-PCR demonstrated that H19 was downexpressed in BMSCs after the transfection of the sh-H19 (compared with sh-NC group, Figure 4A). ORO staining showed that H19 knockdown enhanced lipid droplet formation in BMSCs (compared with Adipogenic+MT+sh-NC group, Figure 4B). Besides, H19 knockdown significantly promoted the expression of CEBPA, CEBPB, CEBPD, FABP4, and PPARG (compared with Adipogenic+MT+sh-NC group, Figure 4C, 4D). Moreover, ARS staining and ALP activity detection revealed that the down-regulation of H19 further reduced the number of mineralized nodules and ALP activity (Figure 4E, 4F). Western blotting manifested that ALP, BMP2, OCN, OPN and Runx2 were down-regulated after H19 knockdown compared with the OS+MT+sh-NC group (Figure 4G). Finally, we probed the influence of H19 on the expression of APN and Wnt/β-catenin pathway under Adipogenic differentiation, and it was found that down-regulating H19 reduced the APN and Wnt/β-catenin expression (compared with Adipogenic +MT+sh-NC group, Figure 4H). Therefore, the above data confirmed that H19 downexpression promoted the Adipogenic differentiation and suppressed osteogenic differentiation of BMSCs.

### miR-541-3p weakened the osteogenic effect of MT-mediated BMSCs

**Figure 5:**
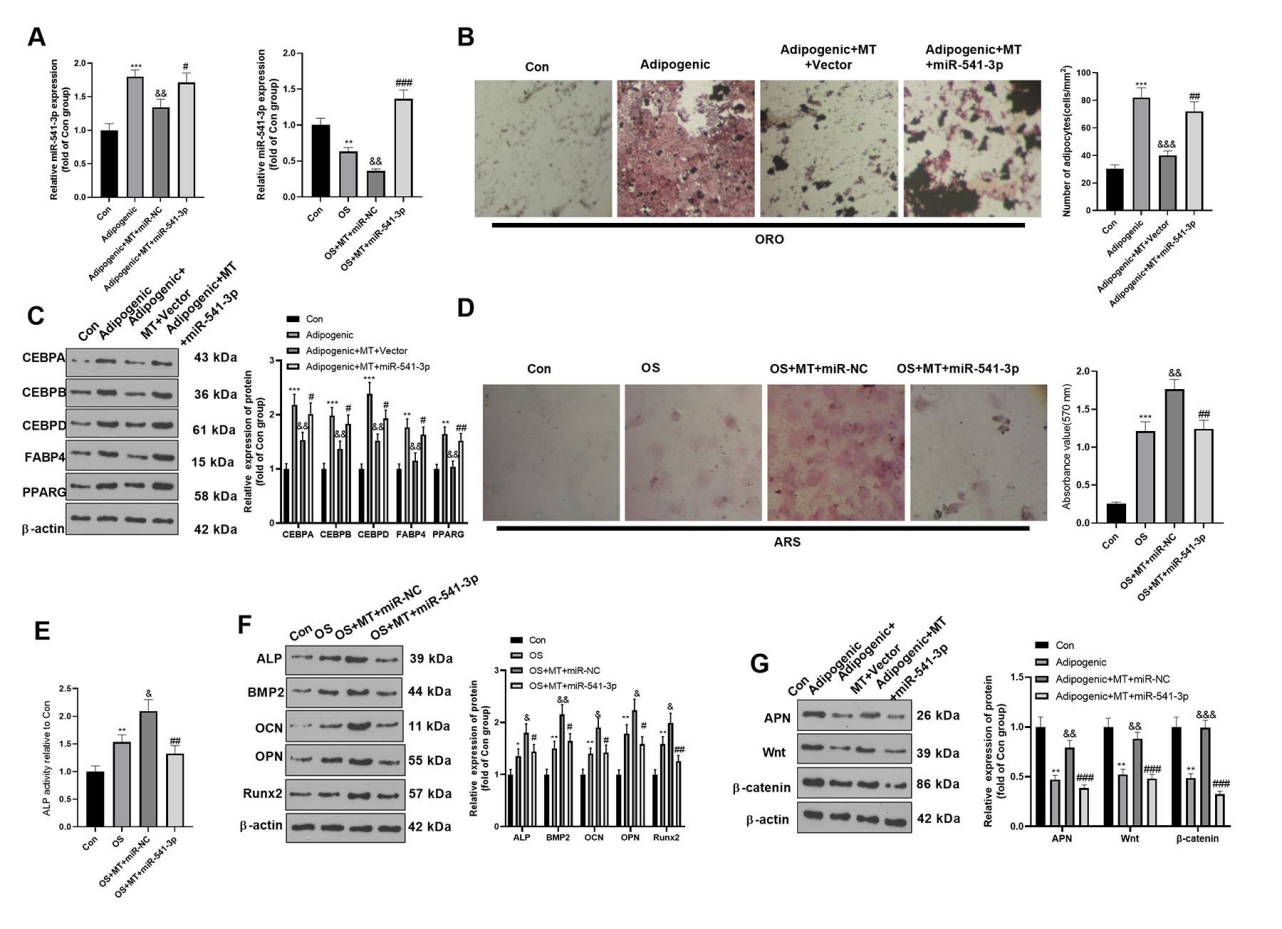
**miR-541-3p weakened the osteogenic effect of MT on BMSCs.** BMSCs were transfected with miR-541-3p or miR-NC, and then cultured in Adipogenic/OS differentiation culture medium. (**A**) qRT-PCR was performed to monitor the miR-541-3p expression BMSCs. (**B**) ORO staining verified the role of H19 in adipogenic differentiation of BMSCs. Scale: 200 μm. (**C**) The expression of adipocyte-related proteins (including CEBPA, CEBPB, CEBPD, FABP4, and PPARG) in BMSCs was analyzed by WB. (**D**) ARS activity test was conducted to evaluated the osteogenic differentiation of BMSCs. Scale: 200 μm. (**E**) The ALP activity was detected using ALP activity test kit. (**F**) The relative expression of osteogenic proteins (including ALP, BMP2, OCN, OPN and Runx2) was analyzed by WB. (**G**) WB was utilized to analyze the protein levels of APN/Wnt/β-catenin in BMSCs cultured in adipogenic differentiation culture medium. *P<0.05, **P<0.01, ***P<0.001 (vs. Con group), &*P*>0.05, &&*P*<0.01, &&&*P*<0.001 (vs. Adipogenic/OS group), #*P*<0.05, ##*P*< 0.01, ###*P*<0.001 (vs. OS+MT+miR-NC). Data were presented as mean ±SEM (n =3) and analyzed using one-way analysis of variance.

The miR-541-3p expression reduced promoted in MT-treated BMSCs. To further confirm the role of miR-541-3p on MT-treated BMSCs, we transfected miR-541-3p mimics and miR-NC in BMSCs to further verify whether miR-541-3p mediated BMSC differentiation. qRT-PCR verified that miR-541-3p was highly expressed in adipogenic/osteogenic differentiated BMSCs (Figure 5A). ORO staining verified that compared with the Adipogenic+MT+miR-NC group, up-regulating miR-541-3p increased lipid droplet formation (Figure 3B) and increased the expression of adipogenesis-related proteins (including CEBPA, CEBPB, CEBPD, FABP4, and PPARG) in BMSCs (Figure 5C). BMSCs transfected with miR-541-3p showed fewer calcium nodules and reduced ALP activity compared with that of the OS+MT+miR-NC group (Figure 4D, 4E). Consistently, Western blotting illustrated that the expression of osteogenic genes (including ALP, BMP2, OCN, OPN and Runx2) in BMSCs was repressed after up-regulating miR-541-3p in adipogenic differentiated BMSCs (Figure 5F), accompanied by the decrease of APN and Wnt/β-catenin expression (Figure 5G). Overall, miR-541-3p weakened the osteogenic effect and increased the adipogenic effect of MT on BMSCs.

### miR-541-3p contained the binding sites of H19 and APN mRNA

**Figure 6:**
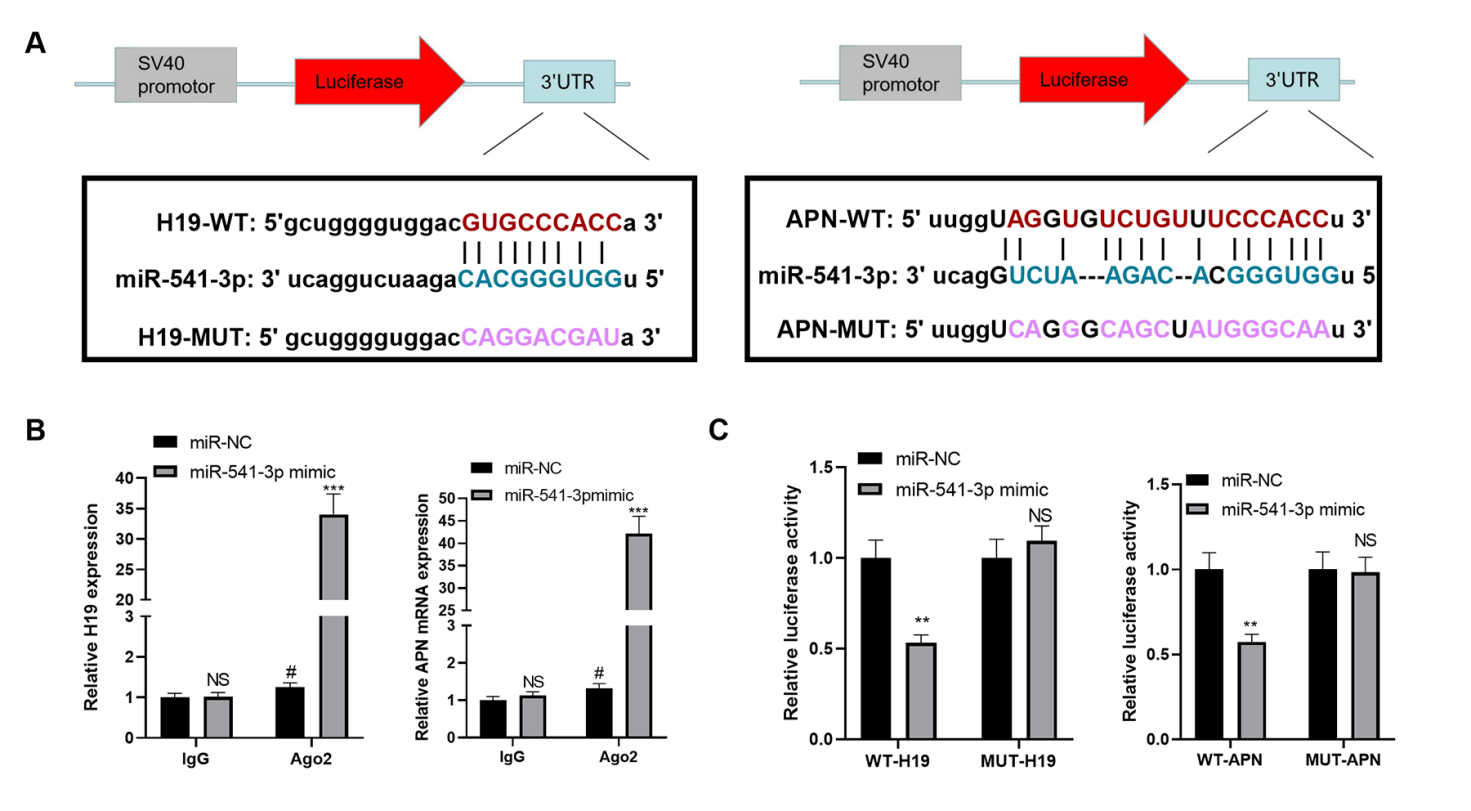
**miR-541-3p contained the binding sites of H19 and APN mRNA.** (**A**) The binding sites between miR-541-3p, H19 and APN mRNA were shown. (**B**) BMSCs were transfected with miR-541-3p or miR-NC, then the RIP experiment was performed to explore the correlation between miR-541-3p and H19, miR-541-3p and APN mRNA. The enrichment of H19, miR-541-3p and APN mRNA were determined by qRT-PCR. NS *P*>0.05, # P<0.05 (vs. miR-NC group), ***P<0.001(vs. IgG group). (**C**) BMSCs was transfected with WT-H19/MUT-H19 or WT-APN/MUT-APN and miR-541-3p or miR-NC. The dual-luciferase reporter assay was implemented to verify the association between miR-541-3p and H19 and APN mRNA. NS *P*>0.05, **P<0.01 (vs. miR-NC group). Data were presented as mean ±SEM (n =3) and analyzed using one-way analysis of variance.

We searched the upstream and downstream genes of miR-541-3p by the bioinformatics database Starbase (https://web.archive.org/web/20110222111721/http://starbase.sysu.edu.cn/) to further explore the upstream and downstream mechanisms of miR-541-3p. It was found that H19 targeted miR-541-3p, while the latter targeted APN mRNA (Figure 6A). In order to clarify the targeting relationship between these three, we conducted a RIP experiment. The results demonstrated that the transfection of miR-541-3p mimics elevated the amount of H19 and APN mRNA precipitated in the Ago2 antibody group (vs. the IgG group), suggesting that H19 and APN were combined with Ago2 through miR-541-3p (Figure 6B). Furthermore, a dual-luciferase reporter assay was implemented to determine the correlation between the three. As a result, miR-541-3p significantly abated the luciferase activity of H19-WT and APN-WT but had little impact on that of H19-MUT and APN-MUT (Figure 6C). These two experiments confirmed that there were binding relationships between miR-541-3p and H19 as well as between miR-541-3p and APN mRNA.

### 3.7 H19 affected the osteogenic and adipogenic differentiation of MT on BMSCs by inhibiting miR-541-3p

**Figure 7:**
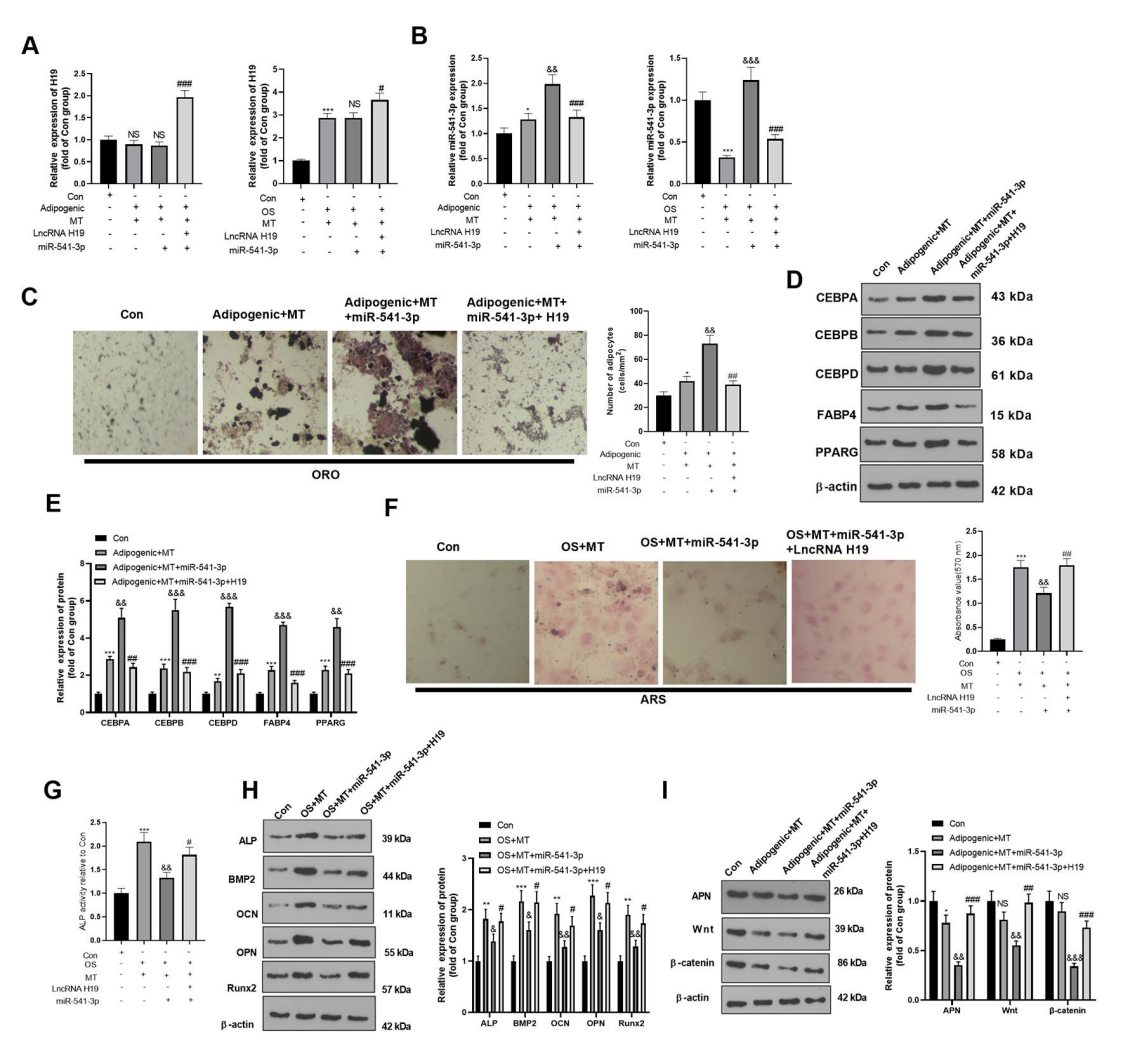
**H19 affected the MT-induced osteogenic and adipogenic differentiation of BMSCs by inhibiting the miR-541-3p/APN axis.** H19 overexpression plasmids and miR-541-3p mimics were transfected into BMSCs, which were cultured in adipogenic/osteogenic differentiation medium and treated with MT (100 μM). (**A**, **B**) The H19 and miR-541-3p expression in adipogenic/osteogenic BMSCs was monitored by qRT-PCR. (**C**) ORO staining verified the role of H19 in adipogenic differentiation of BMSCs. Scale: 200 μm. (**D**, **E**) The expression of adipocyte-related proteins (including CEBPA, CEBPB, CEBPD, FABP4, and PPARG) in BMSCs was analyzed by WB. (**F**) ARS activity test was conducted to evaluated the osteogenic differentiation of BMSCs. Scale: 200 μm. (**G**) The ALP activity was detected using ALP activity test kit. (**H**) The relative expression of osteogenic proteins (including ALP, BMP2, OCN, OPN and Runx2) was analyzed by WB. (**I**) WB was utilized to analyze the protein levels of APN/Wnt/β-catenin in BMSCs cultured in adipogenic differentiation culture medium. *P<0.05, **P<0.01 (vs. Con group), &*P*<0.05, &&*P*<0.01, &&&*P*<0.001 (vs.Adipogenic/OS+MT group), #*P*<0.05, ##*P*<001, ###*P*<0.01 (vs. Adipogenic/OS +MT+miR-541-3p). Data were presented as mean ±SEM (n =3) and analyzed using one-way analysis of variance.

The H19 overexpression plasmids and/or miR-541-3p mimics were co-transfected into adipogenic/ osteogenic differentiated BMSCs. The expression of H19 and miR-541-3p was evaluated. As shown in Figure 7A, 7B, the H19 was overexpressed in the Adipogenic/OS +MT+H19+miR-541-3p group (compared with Adipogenic/OS+MT+miR-541-3p group, Figure 7A). However, miR-541-3p was reduced in the Adipogenic/OS +MT+H19+miR-541-3p group (compared with Adipogenic/OS+MT+miR-541-3p group, Figure 7B). ORO staining showed that up-regulating H19 reduced lipid droplet formation in BMSCs (compared with the Adipogenic+MT+miR-541-3p group, Figure 7C) and repressed the protein levels of CEBPA, CEBPB, CEBPD, FABP4 and PPARG (Figure 7D, 7E). The ARS and ALP activity detection illustrated that the calcium nodules and ALP activity in BMSCs transfected with miR-541-3p mimics decreased, while H19 overexpression reversed these effects (Figure 7F, 7G). At the same time, ALP, BMP2, OCN, OPN and Runx2 were down-regulated in BMSCs after the miR-541-3p mimic intervention, while H19 overexpression reversed miR-541-3p-mediated effects (Figure 7H). Finally, we tested the APN and Wnt/β-catenin expression in adipogenic differentiated BMSCs. It was discovered that compared with the OS+MT+miR-541-3p group, APN and Wnt/β-catenin were up-regulated after up-regulating H19 (Figure 7I). These results suggested that up-regulating miR-541-3p weakened the miR-541-3p up-regulation-induced adipogenic differentiation.

## DISCUSSION

Emerging reports have manifested that MT facilitates osteoblast differentiation [29–31]. In the present research, we confirmed the molecular mechanism of MT on osteogenic and adipogenic differentiation of BMSCs *in vitro* and *in vivo*. The results illustrated that MT up-regulated H19 and inhibited miR-541-3p from activating APN/Wnt/β-catenin pathway, thereby dampening the adipogenic differentiation and enhancing the osteogenic differentiation of BMSCs. As far as we know, this is the first study on the function of MT in osteogenesis by regulating the H19/miR-541-3p/APN axis.

LncRNA H19 is among the most abundant and conserved non-coding transcripts during mammalian development, which is widely involved in the process of osteogenic differentiation of stem cells and contributes to maintaining the osteogenic process of cells [32]. Several studies have demonstrated that H19 is highly expressed during the induction of osteoblastic differentiation of MSCs, and it promotes osteoblastic differentiation by regulating the Wnt/β-catenin activation through miR-141/miR-22 [33]. The up-regulation of H19 and miR-675 abates Smad3 phosphorylation and up-regulates the osteogenesis-related gene Runx2, thereby repressing the adipogenic differentiation of BMSCs and promoting osteoblastic formation [34, 35]. H19 can also target DKK4 to activate the Wnt/β-catenin signaling, thereby improving OP [36]. Previously, some studies have illustrated that MT is implicated in regulating the H19 expression, and MT treatment blocks the senescence of c-kit (+) cardiac progenitor cells through the H19/miR-675/USP10 signaling axis [37]. Additionally, MT treatment can protect the nervous system by improving the transcription efficiency of H19 during early brain injury [38]. However, whether MT and H19 contribute to OP’s pathogenesis and whether they participate in the regulatory mechanism of osteogenic and adipogenic differentiation balance of BMSCs has not been studied yet. In this study, H19 mRNA was found to be up-regulated in BMSCs in the presence of MT. Functional tests showed that H19 overexpression significantly decreased the expression of adipogenesis-related genes and increased the expression of osteogenesis-related genes, suggesting that H19 mediates the osteogenic effect of MT on BMSCs.

MicroRNAs (miRNAs) are small, double-stranded non-coding RNAs with 20-25 nucleotides in length. They can regulate gene expression at the post-transcriptional level by inhibiting messenger RNA (mRNA) translation or promoting mRNA degradation. MiRNAs are powerful regulators of various cell activities (including cell growth, differentiation, development and apoptosis) [39, 40]. Multiple studies have confirmed that miRNAs regulate osteogenic and adipogenic differentiation of BMSCs [41]. Additionally, various miRNAs, including miR-155 [42], miR-223 [43], and miR-16-5p [44], contribute to MT-mediated biological functions. Recently, Wu et al. demonstrated that lncRNA FAM83H-AS1 contributed to osteogenic differentiation of staphylococcal protein A (SpA)-induced hBMSCs through negatively mediating miR-541-3p [45]. Nevertheless, the function of miR-541-3p in the osteogenesis protection of MT on BMSCs remains unclear. This study revealed that miR-541-3p was repressed in MT-treated BMSCs, and overexpressing miR-541-3p suppressed the osteogenic effect and increased the adipogenic effect of BMSCs mediated by MT, which verified that miR-541-3p had an inhibitory effect on the osteogenesis of MT-mediated BMSCs.

Increasing studies have revealed that lncRNAs regulate miRNAs as competing endogenous RNAs (ceRNAs). For instance, lncRNA XIXT up-regulates RUNX2 by sponging miRNA-30a-5p, thereby inducing osteogenesis of hBMSCs and alleviating OP [46]. As a ceRNA, KCNQ1OT1 actively regulate the osteogenic differentiation of BMSCs by sponging miR-214 to modulate BMP2 [47]. Our study confirmed the expression and biological functions of H19 and miR-541-3p in BMSCs, while the interaction between them remains unclear. Therefore, we conducted the dual-luciferase reporter gene assay and RIP analysis. The results showed that H19 bound to miR-541-3p, and their expression was negatively correlated. Overexpressing H19 significantly abated the miR-541-3p expression in BMSCs and enhanced the effect of MT on BMSCs by inhibiting miR-541-3p.

Many signaling pathways are found involved in MT-mediated differentiation of BMSCs. For example, Metformin promoted osteogenic differentiation of hBMSCs through inhibiting phosphorylation of GSK3β via activating AMPK and Wnt signaling pathway [48]. In another study, the implant osseointegration in a rat model was promoted by Metformin dependently through AMPK/BMP/Smad signalling pathway [49]. As an adipocyte-specific factor, APN has been found to promote osteogenic differentiation of stem cells. For example, human amnion-derived mesenchymal stem cells (HAMSCs) promoted the growth, osteoblastic differentiation, and APN excretion in human adipose-derived stem cells (HASCs) through leucine zipper motif (APPL1) induced extracellular signaling-regulated kinase 1/2 (ERK1/2) phosphorylation [50]. What’s more, several studies confirm that APN activates Wnt signaling pathway [28, 51]. The expression level of APN in organisms is regulated by multiple factors, among which miRNAs are important regulators of APN secretion in adipose tissues, such as miR-876-3p, miR-193b, etc. [52, 53]. Here, we discovered that APN was heightened, and overexpressing miR-541-3p decreased APN expression in MT-treated BMSCs. At the same time, we discovered that H19 was positively correlated with APN expression in BMSCs, and APN expression was significantly elevated after overexpressing H19 in BMSCs. These findings suggested that MT affected the osteogenic differentiation of BMSCs through H19/miR-541-3p/APN in OP.

Overall, MT increases the osteogenic differentiation and abates the adipogenic differentiation of BMSCs by acting on the H19/miR-541-3p/APN pathway, providing a new perspective for studying the pharmacological effect of MT in the treatment of OP.

## MATERIALS AND METHODS

### Animal

Thirty Sprague Dawley (SD) female rats (eight-week-old, 220-250 g) were bought from the Animal Experimental Center of Shandong University. All the rats were kept in cages. The rats were fed with food and water with 12-hour periods of light (light intensity: 30-45 micro/cm^2^) and darkness, respectively (20- 25° C, 50% to 52% humidity). All experiments were approved by the ethics committee of Jinan Stomatological Hospital (approve number: JNSH-2019-032) and were in accordance with the guidelines of the National Institutes of Health on animal care and use.

### Animal grouping and model establishment

Thirty SD rats were randomized into the sham operation group and model group (15 rats/group). The model group was anesthetized with 2% pentobarbital (drug concentration was 40 mg/kg). After skin preparation, the rats were fixed in the supine position and disinfected with 75% alcohol. The back skin and peritoneum of the rats were cut open to expose ovaries. After ovariectomy, ligation was performed with absorbable suture, and back skin was sutured. In the sham operation group, the anesthesia, fixation, and selected incision were the same as those in the model group. However, the ovaries were preserved, and only the surrounding fat was removed. The rats in the two groups were intraperitoneally injected with penicillin (2.0×10^5^ IU/kg) for three days after the operation to prevent infection. They were then caged for feeding. The bone tissue of the mandible was excised and fixed in 4% formaldehyde for subsequent histological analysis. Osteoclasts were labeled by Tartrate-resistant acid phosphatase (TRAP) staining.

### Micro-CT analysis

CT detection was performed by referring to the previous method [26]. Briefly, after euthanasia, the mandibular bone tissue of experimental rats was collected and immobilized in formalin saline solution immediately. Micro-CT scanner (Scco μCT 80, Scanco Medical AG, Bassersdorf, Switzerland) with a 16 μm voxel size was utilized to assess the structural parameters of the trabecula and cortical region, as well as the cortical region of the mandible. The parameters, including trabecular BMD, BV/TV, trabecular thickness (Tb.Th), trabecular spacing (Tb.Sp) and Tb.N, were calculated by standard 3D microstructural analysis.

### Enzyme-linked immunosorbent assay (ELISA)

1 mL of rat tail vein blood samples were collected, placed overnight at 4° C, and centrifuged (1000 rpm, 20 min). The supernatant was then taken. The MT expression in the rat serum (50 μL) in each group was determined by the ELISA Kit (USCNK, USA) following the kit instructions.

### Acquisition and culture of BMSCs

After the modeling, the rat mandible was separated under aseptic conditions. The mandible was cut off with bone scissors, and the complete medium containing 10% fetal bovine serum (FBS) was extracted with a syringe to flush out the bone marrow. The cells were dissociated, suspended (1×10^5^ cells/mL), and inoculated in the culture bottle, which was incubated at 37° C with 5% CO_2_ and 95% humidity (Thermo, USA). After 48 hours, the whole solution was changed, and then the solution was changed every three days. After about ten days, the cell colonies gradually covered the bottom of the culture flask, and the cell trypsinization and sub-culture were performed. BMSCs were treated with 100 μmol/L MT, and the impact of MT on adipogenic/osteogenic differentiation of BMSCs was probed. The MT2 selective inhibitor 4-P-PDOT (1 μg/ml) (R&D Systems, Tocris Bioscience, Cat. #1034) was administered into the BMSCs for inhibiting MT2.

### Cell transfection

BMSCs were cultured in the DMEM (Hyclone, Logan, USA) containing 10% FBS (Gibco, NY, USA), 100 U/mL penicillin and 100 mg/mL streptomycin with 5% CO_2_ at 37° C. H19 overexpression plasmids, H19 negative vectors, miR-541-3p mimics and miR-NC were all provided by GenePharma (Shanghai, China). sh-H19 and its negative control sh-NC were purchased from RiboBio (Guangzhou, China). They were transfected into BMSCs with Lipofectamine®3000 (Invitrogen; ThermoFisherScientific, Inc.) according to the instructions of the manufacturer.

### Adipogenic differentiation

When the cell fusion rate reached 80%-90%, the cells were cultured in the adipogenic differentiation medium (AM) (Cyagen Biosciences, USA) for 16 days. AM A was composed of 175 mL medium, 10% FBS, 1% penicillin-streptomycin, 1% glutamine, 0.1% dexamethasone, 0.2% insulin, 0.1% rosiglitazone, and 0.1% isobutyl methyl xanthine (IBMX). In contrast, AM B contained 175 mL medium, 10% FBS, 1% glutamine, 1% penicillin-streptomycin and 0.2% insulin. BMSCs were cultured in AM A for three days and then in AM B for one day to induce adipocytes.

### Osteogenic differentiation

BMSCs were cultured in 6-well plates at 37° C with 5% CO_2_ under standard culture conditions. Cells were stored in a normal medium (Kane) until the fusion rate reached 80%. Then, they were differentiated into osteoblasts using the osteoblastic differentiation (OD) induction medium (Kane), which was supplemented with 175 mL medium, 10% FBS, 1% glutamine, 1% penicillin-streptomycin, 0.2% ascorbic acid, 1% β-glycerophosphate and 0.01% dexamethasone, for 14 days. The medium was changed every three days.

### Oil red O (ORO) staining

ORO staining was employed to test lipid droplet formation of BMSCs after adipogenic differentiation [27]. After fat formation, the cells were washed three times with PBS (Solarbio, China). They were then immobilized at room temperature in 4% paraformaldehyde (PFA, SolarBio, China) for 30 min. After washing with PBS, the cells were stained with ORO staining solution (Cyagen Bioscience, USA) for 20 min. Finally, ten random images were taken using an inverted light microscope (Nikon, Japan). Under a microscope, fat cells showed red oil droplets. We calculated the number of fat cells per square millimeter (mm^2^).

### Alizarin red S (ARS) staining

BMSCs were cultured for 13 days and immobilized with 4% PFA for 20 min. They were washed with phosphate buffer solution (PBS) and dyed with 0.1% ARS (Sigma-Aldrich, USA) for 5-10 min. Thirty minutes later, the formation of calcified nodules was observed under an inverted microscope. Then the dye was extracted with 10% cetylpyridinium chloride (CPC; Sigma), and the optical density at 570 nm was measured.

### Determination of alkaline phosphatase (ALP) activity

After incubation for seven days, the cell lysates were extracted with 1% Triton X-100 on ice for 30 min. They were then centrifugated (12000 rpm) at 4° C for 5 min, and the ALP activity was monitored following the manufacturer’s instructions (Beyotime Biotechnology Co., Ltd., Shanghai) based on the concentration of phenol in the standard well. The adjustment was made according to the protein content in each sample.

### Quantitative reverse transcription-PCR (qRT-PCR)

Total RNA was isolated from mandibular tissues and cells with the TRIzol reagent and reversely transcribed into cDNA with the PrimeScript™ RT Reagent kit (Invitrogen, Shanghai, China). qRT-PCR was conducted using the Bio-Rad CFX96 quantitative PCR system and SYBR, with an initial denaturation at 95° C for 5 min, denaturation at 95° C for 15 s, and annealing at 60° C for 30 s. GAPDH was the endogenous control of H19, while U6 was that of miR-541-3p. The 2^(-ΔΔCt)^ method was used for statistics. Each experiment was conducted three times. Specific primer sequences were shown in Table 1.

**Table 1:** Primers used in this study.

**The target**	**Forward (5 '-3')**	**Reversion (5 '-3')**
LncRNA H19	TCTTGCTCTTTCTGCCTGGA	GAGGTTTAGGGGATCGAGGG
miR-541-3p	AACAAGTGGTGGGCACAGAATC	CAGTGCAGGGTCCGAGGT
U6	CTCGCTTCGGCAGCACA	AACGCTTCACGAATTTGCGT
GAPDH	GGGAGCCAAAAGGGTCAT	GAGT CCTTCCACGATACCAA

### Western blotting

The RIPA lysis buffer containing protease inhibitors (Beyotime Biotechnology, Shanghai, China) was utilized to extract proteins from mandible tissues and cells, and the BCA kit (San Jose, USA) was employed to examine the content of isolated proteins. After SDS-polyacrylamide gel electrophoresis, the proteins were transferred to cellulose nitrate membranes, which were then blocked with 5% skim milk for one hour and incubated with the antibodies of BMP2 (1:1000, ab214821, Abcam, MA, USA), Runx2 (1:1000, ab236639), OPN (1:1000, ab8448), OCN (1:1000, ab133612), CEBPA (1:1000, ab40761), CEBPB (1:1000, ab32358), CEBPD (1:1000, ab245414), FABP4 (1:1000, ab92501), PPARG (1:1000, ab178860), ALP (1:1000, ab229126), APN (1:1000, ab181281), Wnt (1:1000, ab219412), β-catenin (1:1000, ab32572), and β-actin (1:1000, ab8227) overnight at 4° C. After washing, the membranes were incubated with peroxidase-bound secondary antibodies for one hour at room temperature. Finally, the bands were developed with an ECL kit (Amersham Pharmacia Biotech, Little Chalfont, UK).

### RNA immunoprecipitation (RIP) assay

In brief [28], RIP analysis was performed using the Magna RIP Kit (Macquarie, USA) and Ago2 antibody (Cell Signaling Technology, USA). Briefly, 10^7^ transfected cells were washed twice in cold PBS, lysed in an equal volume of RIP lysis buffer, and then incubated with 5 μg primary antibody at 4° C for 2 hours. After that, 50 μL prepared magnetic bead suspension was added to each sample and incubated overnight at 4° C. The beads were briefly washed five times with RIP buffer and then resuspended in 500 μL TRIzol LS (Life Technologies). The contents of H19, APN mRNA and miR-541-3p in lysates were determined by qRT-PCR.

### Dual-luciferase reporter assay

The dual-luciferase reporter assay was adopted to test the targeted association between the miR-541-3p family and the 3’-untranslated region (3'-UTR) of H19 or APN. The wild-type (WT) H19 sequence or the WT 3'-UTR fragment of APN mRNA was amplified and inserted into the pmiRGLO dual-luciferase miRNA target expression vector (Promega Corp., Madison, WI, USA) to construct pmiRGLO-H19-WT or pmiRGLO-APN-WT. The GeneArt™ Site-Directed Mutagenesis PLUS System (cat. no. A14604; Thermo Fisher Scientific, Inc.) was adopted to mutate the putative binding site of the miR-541-3p family in H19 or APN 3’-UTR. MUT H19 or APN 3 '-UTR was inserted into the pmiRGLO vector to form pmiRGLO-H19-MUT or pmiRGLO-APN-MUT. The corresponding reporter vector and miR-541-3p mimic or NC mimic were co-transfected into BMSCs and incubated for 48 hours. The luciferase activity was monitored with the Dual-Luciferase Reporter Assay System (Promega Corp.).

### Statistical analysis

The student’s *t* test was employed to compare the differences between the two groups. Pearson correlation analysis was adopted to determine the correlation between H19 and miR-541-3p, MT, APN, and BMD in the bone tissue of OP rats. The Tukey-Kramer test was used to conduct a one-way analysis of variance for multiple groups of data. All results were expressed as mean ±SEMS. The experiment was carried out in triplicate. The GraphPad Prism software (version 8.0) was utilized for the drawing. *P*<0.05 indicated statistical significance.

### Ethics statement

Our study was approved by the Animal Ethics committee of Jinan Stomatological Hospital (approve number: JNSH-2019-032).

### Data availability statement

The data sets used and analyzed during the current study are available from the corresponding author on reasonable request.
